# Effect of Intrinsic Ripples on Elasticity of the Graphene Monolayer

**DOI:** 10.1186/s11671-015-1135-5

**Published:** 2015-10-26

**Authors:** Seungjun Lee

**Affiliations:** Department of Mechanical, Robotics and Energy Engineering, Dongguk University-Seoul, Seoul, 100715 Korea

**Keywords:** Graphene, Ripples, Elasticity, Molecular dynamics

## Abstract

The effect of intrinsic ripples on the mechanical response of the graphene monolayer is investigated under uniaxial loading using molecular dynamics (MD) simulations with a focus on nonlinear behavior at a small strain. The calculated stress-strain response shows a nonlinear relation through the entire range without constant slopes as a result of the competition between ripple softening and bond stretching hardening. For a small strain, entropic contribution is dominant due to intrinsic ripples, leading to elasticity softening. As the ripples flatten at increasing strain, the energetic term due to C–C bonds stretching competes with the entropic contribution, followed by energetic dominant deformation. Elasticity softening is enhanced at increased temperature as the ripple amplitude increases. The study shows that the intrinsic ripple of graphene affects elasticity. This result suggests that a change of ripple amplitudes due to various environmental conditions such as temperature, and substrate interactions can lead to a change of the mechanical properties of graphene. The understanding of the rippling effect on the mechanical behavior of 2D materials is useful for strain-based ripple manipulation for their engineering applications.

## Background

Graphene has received considerable interest due to its unique properties, potentially leading to a wide range of applications such as display screens [1, 2], energy storage [3–5], solar cells [6–8], and field-effect transistors [9–11]. As one of its unique properties, suspended graphene is not perfectly flat but forms spontaneous ripples on the surface [12]. Thermal fluctuation is known for the origin of out-of-plane deformations, and the intrinsic ripples are inevitable to maintain the stability of 2D crystal structures [13]. The spontaneous roughening of graphene is intriguing because it is attributed to unexpected physical characteristics such as a negative thermal expansion coefficient [14], increasing bandgap [15], and unusual electronic properties [16].

The mechanical properties of graphene have been widely studied experimentally and theoretically. In terms of experimental studies, Lee et al. performed a pioneering mechanical test to measure the Young’s modulus and fracture strength of a monolayer graphene membrane, reporting the values as 1 TPa and 130 GPa, respectively [17]. Since direct measurement of the mechanical properties is challenging for the atomically thin membrane, they used a nanoindentation technique on a freely suspended graphene membrane using an atomic force microscope. Recently, a direct uniaxial tensile test was performed with a micromechanical device to measure the fracture toughness of graphene with a pre-crack [18]. In terms of theoretical studies, various atomistic calculations using density functional theory (DFT) [19], molecular dynamics simulations and tight-binding (TB) approach [20] were performed by adapting the traditional uniaxial tension test.

Theoretical studies usually predict higher mechanical strength since they assume a perfect crystalline structure while experimentally synthesized samples often have defects and grain boundaries [21]. To complete this discrepancy, systematic simulations have been widely performed, focusing on the effect of imperfection such as grain boundaries [22], Stone-Wales defects [23], and nanopores [24]. Expanding the single defect issue, the effect of a large number of defects has recently been researched [25, 26]. The uniaxial tensile test of highly defective graphene showed totally different mechanical responses such as the lowering of Young’s modulus and ductile fracture behavior [26]. In addition, Zhang et al. obtained the stress-strain curve of nanocrystalline graphene from a tension test, showing a clear nonlinear behavior at the initial stage [27]. They showed that the nonlinear mechanical behavior at the beginning is attributed to entropic contribution due to wrinkles produced by atomic mismatches at grain boundaries. This interesting behavior induced by out-of-plane deformations raises questions about whether a similar characteristic also exists in the defect-free pristine graphene monolayer since it has intrinsic ripples. However, although the stress-strain relation of pristine graphene has been obtained in many theoretical studies, the nonlinear elasticity at a small strain has rarely been mentioned.

The nonlinear stress-strain relation of graphene has been proposed in various experimental studies [17, 28]. However, in these studies, the experiment focused on the overall response at a large strain. Furthermore, most of the experiments measure the stress-strain relation using an indentation technique, which does not effectively capture the rippling effect since the indentation direction is same as the out-of-plane displacements: the ripple amplitudes may change due to the interaction with the indentation tip. Theoretically, while ab initio calculation reported that linear-elastic behavior lasts up to 5 % strain, the rippling effect was not considered, assuming only the in-plane deformation [29]. Since it is difficult to describe out-of-plane deformations in the DFT studies, molecular dynamics (MD) simulation is an effective technique to study the effect of ripples under axial loading. Although many MD simulations are performed to study the mechanical behavior of graphene, the detail studies on the mechanical behavior are rarely attended, especially for small strain regions. For example, the Young’s modulus of graphene is typically calculated by assuming a linear trend in the stress-strain curve at a small strain [20]. However, questions still remain: how far a linear region can be defined in the curve for the slope fitting, whether a linear region exists at infinitesimal strain, and how the intrinsic ripples in pristine graphene affect the mechanical behavior. The effect of the intrinsic ripples on the mechanical properties is nontrivial because the ripple amplitudes of the graphene on a substrate for an engineering application can change according to the surface properties such as roughness and interfacial van der Waals interactions [30].

In the paper, the mechanical behavior of a suspended monolayer graphene is investigated under uniaxial stretching using 3D and 2D MD simulations, focusing on the effect of intrinsic ripples. The study revealed that the linear region does not exist even at a small strain and the out-of-plane displacements result in elasticity softening at a finite temperature. This paper is structured as follows. First, the MD simulation model and methods for uniaxial tension are described. Then, the mechanical response of a monolayer graphene is investigated and the effect of ripples is studied with varying temperature. Finally, the conclusion is given at the end.

## Methods

**Fig. 1:**
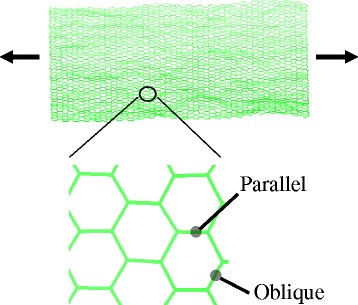
Equilibrated monolayer graphene showing intrinsic ripples

**Fig. 2:**
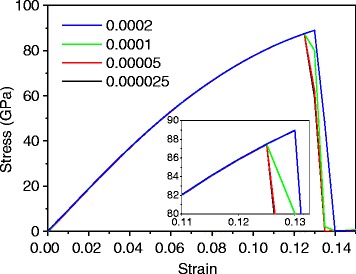
Stress-strain response at different strain rates of 0.0002 (*blue*), 0.0001 (*green*), 0.00005 (*red*), and 0.000025 (*black*)

All MD simulations are performed using the open-source program LAMMPS [31]. The second generation reactive empirical bond order (REBO) potential [32] is used unless it is mentioned. A square-shaped monolayer graphene is simulated with periodic boundary conditions. Figure 1 shows the equilibrated graphene monolayer for a tensile test. The length of one side is selected as 100 Å, which is large enough to ignore the size effect of graphene [20]. In the *z* direction, a large height for the simulation box is given to remove the graphene interlayer interactions. A time step of 1 fs is used. The Nose-Hoover thermostat and Nose-Hoover barostat are used in all simulations with a temperature damping constant of 0.1 ps and pressure damping constant of 1 ps. The system is equilibrated with the NPT ensemble at zero pressure to remove any internal stress. After equilibrium, a uniaxial tension test is performed with the NVT ensemble. The uniaxial loading is applied by enlarging the simulation box with a strain rate of 5 × 10^−5^/ps. Figure 2 shows the stress responses at different strain rates. The overall responses are similar except fracturing. The magnified view at fracture is shown in the inset figure. The fracture behavior is converged below a strain rate of 5 × 10^−5^/ps. The strain increment is applied every 100 ps. The atomic stresses are obtained by averaging during the last 10 ps at each increment. Stresses are calculated using the Virial theorem, which is expressed as [33, 34]

1 $$ \upsigma \left(\mathbf{r}\right)=\frac{1}{\Omega}\sum_i\left[-{m}_i{\dot{\mathbf{u}}}_i\otimes {\dot{\mathbf{u}}}_i+\frac{1}{2}\sum_{j\ne i}{\mathrm{r}}_{ij}\otimes {\mathbf{f}}_{ij}\right], $$

where Ω is the total volume, *m*_*i*_ is the mass of atom *i*, ${\dot{\mathbf{u}}}_i$ is the time derivative of **u**_*i*_, **u**_*i*_ denotes the displacement vector of atom *i* relative to a reference position, **r**_*ij*_ = **r**_*j*_ − **r**_*i*_, ⊗ is the cross product, and **f**_*ij*_ is the interatomic force applied on atom *i* by atom *j*. The engineering stress is calculated by applying the volume of the equilibrated system. The length and width for the volume is obtained from the length of the simulation box in the *x* and *y* directions. The thickness is assumed as 3.35 Å. Since the initial volume is used for the stress calculation, the stress obtained in this study is engineering stress. The stress level may change if true stress is calculated. To remove the thermodynamic vibration effect, ten points are chosen randomly in the range of the last 10 ps at the equilibrated state, serving as a starting point of the uniaxial test. The results of ten simulations are averaged. The time average fluctuation height of the graphene surface is calculated by

2 $$ {\langle h\rangle}_t=\sqrt{{\left\langle \sum_{i=1}^N\frac{{\left({h}_i-\overline{h}\right)}^2}{N}\right\rangle}_t}, $$

where 〈〉_*t*_ is the time average, *N* is the total number of atoms, *h*_*i*_ is the *z* direction displacements of *i* atom, and $\overline{h}$ is the spatial average height of atoms.

## Results and Discussion

### Mechanical Response of Graphene Under Uniaxial Tension

**Fig. 3:**
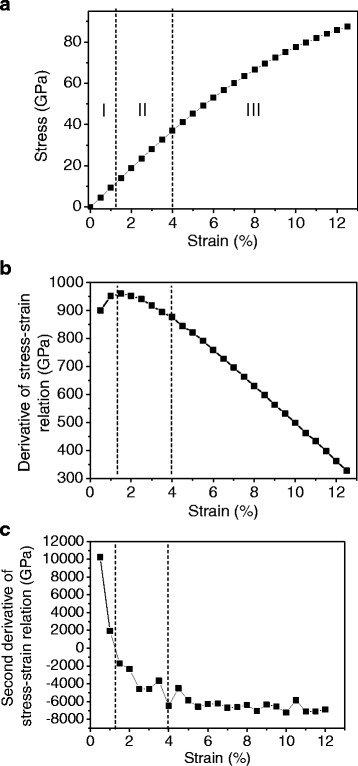
**a** Stress-strain response of a monolayer graphene under uniaxial tensile test in the armchair direction at 300 K. **b** Derivative of stress-strain relation. **c** Second derivative of stress-strain relation

**Fig. 4:**
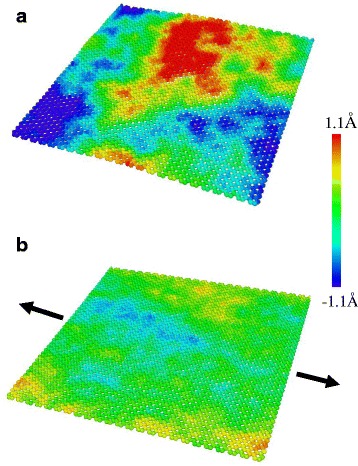
Snapshots of the MD simulations showing the out-of-plane fluctuation of a monolayer graphene at 300 K with (**a**) 0 % strain and (**b**) 0.5 % strain

**Fig. 5:**
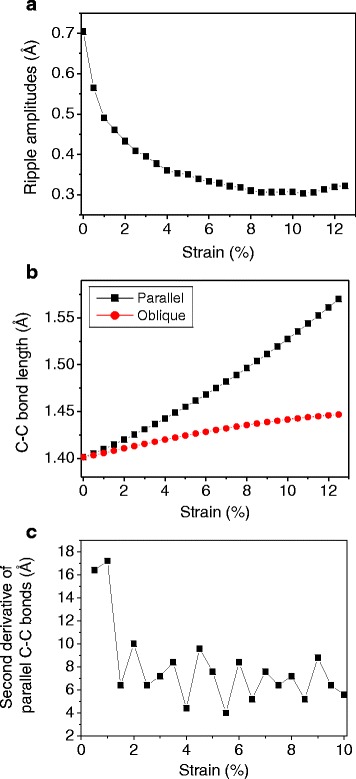
**a** Ripple amplitude variation. **b** C–C bond length (*black squares* the length variation of C–C bonds in the parallel direction to the applied loading; *red dots* the length variation of C–C bonds in the oblique direction) (see Fig. 1). **c** The second derivative of the parallel C–C bonds

Figure 3a shows the stress-strain response of a monolayer graphene at 300 K under uniaxial tension in the armchair direction. The fracture occurs at 0.125 and the fracture stress is 87 GPa, showing good agreement with previous study [20]. While the nonlinear relation is observed clearly over 0.06 strain, it is not clear at small strains. To investigate linearity in detail, the derivative and the second derivative of the stress-strain curve are calculated and shown in Fig. 3b, c. The curve is divided into three regions according to the second derivatives: positive, negative with decreasing, and negative with constant. In the first region, the tangent slope of the curve increases, which is the characteristic of entropic elasticity that is observed in polymer [35] or biological materials [36]. This initial nonlinearity is similar to the behavior shown in polycrystalline graphene, implying that the effect of intrinsic ripples also exists in the pristine graphene. The MD simulation snapshots are shown in Fig. 4. The intrinsic ripples on a graphene monolayer are clearly diminished as strain increases. The ripple amplitude variation upon stretching is shown in Fig. 5a. Initially, the surface has no constraint allowing the out-of-plane displacement of 0.7 Å. However, as strain is applied, the ripple amplitude decreases sharply, indicating that ripple effect disappears rapidly at a small strain.

In the second region, the tangent slopes begin to decrease because the effect of C–C bond stretching emerges, competing with the effect of ripples. The length of the C–C bonds is calculated to investigate whether the C–C bond stretching begins after the ripples flatten or at the same time with the reduction in the ripple amplitude. There are two types of the C–C bonds that are parallel and oblique to the applied loading direction as shown in Fig. 1. Twenty C–C bonds are randomly selected for each type, and their lengths are averaged. The standard deviation of the C–C bonds length for 20 samples is calculated as 0.0002, showing a uniform variation. As shown in Fig. 5b, the C–C bonds are stretched as soon as stretching is applied. However, as seen in the feature of the second derivatives of the C–C bond stretching in Fig. 3c, the bond stretching is nonlinear initially and turns to linear, indicating that ripples affect bond stretching at the initial stage of loading. The initial average ripple amplitude is less than 1 Å, which is comparable to the bonding length. Thus, the uniaxial loading affects simultaneously both the reduction in the ripple amplitude and the increase of the length of C–C bonds, although the effect of bond stretching is not considerably large at the initial stage.

In the third region, the second derivatives are almost constant, indicating quadratic stress-strain behavior. In this region, although the ripple amplitude decreases continuously, the fluctuation reduction is small so the effect of the C–C bond stretching is dominant.

Recently, DFT calculations showed that strain-induced hardening in the out-of-plane acoustic phonon mode explains the absence of rippling in graphene under a biaxial strain [37, 38]. In the study, the variation of the coefficients *A* and *B* in *w*^2^ = *Ak*^4^ + *Bk*^2^ of elasticity model has been calculated as a function of strain [37]. As strain increases, the phonon dispersion curve in the *z* direction changes from quadratic to linear, representing the strain hardening. The strain hardening in the out-of-plane atomic motion results in suppressing the ripple amplitudes. The DFT calculation results are well comparable to our MD simulations although the DFT calculations are studied under a biaxial strain. For example, the crossover of the *A* and *B* variance in the DFT calculation is around 1.7 % strain, which agrees with the strain of the maximum tangent modulus in our MD simulation. In addition, the quadratic relation in the out-of-plane phonon mode changes to linear after 5 % strain in the DFT calculation, while the linear bond stretching occurs after 4 % strain in our MD simulations as shown in Fig. 5c.

**Fig. 6:**
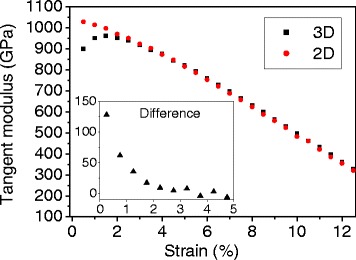
Tangent modulus of 3D (*black squares*) and 2D (*red dots*) MD simulations at 300 K

To confirm the effect of intrinsic ripples on the mechanical behavior, 2D MD simulations are performed. In 2D simulations, no space is given in the *z* direction and the *z*-components of velocities and forces are removed at every time step in order to prevent the atoms from moving in the *z* direction. Figure 6 shows a comparison of tangent moduli between 2D and 3D simulations. Unlike the 3D case, the tangent modulus in 2D continuously decreases from the beginning of the test. To emphasize the effect of ripples, the difference between 3D and 2D tangent moduli is shown in the inset figure. First, the difference is about 130 GPa, which represents the amount of elasticity softening due to ripples. The softening effect reduces rapidly as strain increases, and the difference becomes almost zero after about 4 % strain: the rippling effect is diminished, and the mechanical behavior follows the C–C bond stretching responses. A similar trend between the 2D and 3D difference in Fig. 6 and ripple reduction in Fig. 5a confirms that the nonlinear behavior at a small strain in 3D simulations is due to rippling.

### Effect of Ripples on Graphene Elasticity

**Fig. 7:**
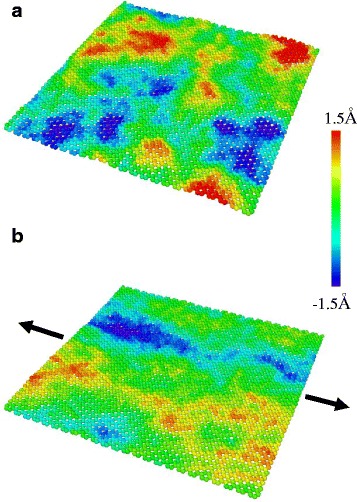
Snapshots of the MD simulations showing the out-of-plane fluctuation of a graphene monolayer at 1000 K with (**a**) 0 % strain and (**b**) 0.5 % strain

To investigate the effect of ripples on graphene elasticity, a series of MD simulations is performed with varying temperature since the ripple amplitude highly depends on temperature. The simulations are conducted using the optimized Tersoff potential proposed by Lindsay and Broido [39] together with the REBO potential. The optimized Tersoff potential predicted better thermal [39] and mechanical [40] properties of carbon-based nanostructures. Selected MD snapshots at 1000 K with the REBO potential are shown in Fig. 7. Similar to the 300 K case in Fig. 4, the overall ripple amplitudes decrease as strain increases. However, at 1000 K, the maximum ripple amplitudes are higher and the shorter wavelength ripples appear with 0 % strain. Notably, as strain increases, randomly distributed ripples at 0 % strain tend to align perpendicular to the direction of the applied strain as shown in Fig. 7b. The ripples in the strain direction are suppressed so the perpendicular ripples remain freely and appear on the surface.

**Fig. 8:**
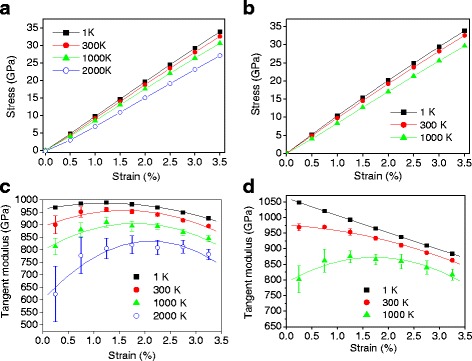
**a** Stress-strain response using the REBO potential. **b** Stress-strain response using the Tersoff potential. **c** Tangent modulus using the REBO potential. **d** Tangent modulus using the Tersoff potential

Here, the mechanical behavior at a relatively small strain is focused because entropic contribution due to rippling is dominant at the initial stage as discussed previously. Selected results are shown in Fig. 8a, b, which are obtained using the REBO and Tersoff potential, respectively. For the Tersoff potential, defects and bond breaking are observed beyond a temperature of 1200 K during equilibration, so the stress responses are calculated up to 1200 K. As the temperature increases, the initial slope of the stress-strain curves decreases, lowering the elastic modulus. In particular, at 2000 K in Fig. 8a, a nonlinear relationship analogous to entropic elastic behavior is shown clearly from the beginning of the curve. To more clearly observe the slope change, the tangent modulus is calculated in Fig. 8c, d. For the REBO potential shown in Fig. 8c, the tangent elastic moduli increase initially and decrease without a constant region after reaching the maximum at all different temperature ranges, even at extremely low temperature (1 K). The large reduction at high temperature is due to the increase of thermal fluctuation, which leads to the enhancement of the entropic contribution. In addition, as temperature increases, the location of the maximum slope moves toward a higher strain because a larger strain is needed to flatten the enlarged ripples at higher temperature. For the Tersoff potential shown in Fig. 8d, the trend at low temperature is different. The initial increase shown in the REBO potential is not observed at 1 and 300 K. The curve of 1 K almost matches with the 2D simulation. The results indicate that the softening effect due to ripples is not significant at low temperature for the Tersoff potential compared to the REBO potential.

**Fig. 9:**
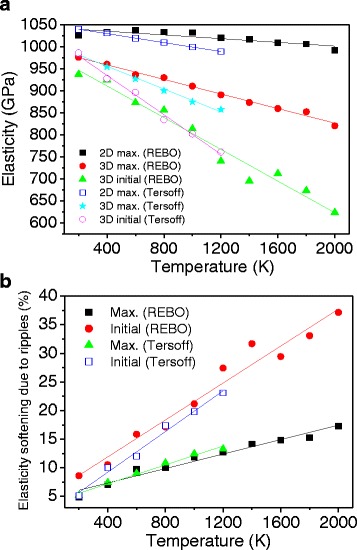
**a** Variation of tangent modulus at increasing temperature: 2D maximum tangent modulus using the REBO potential (*black squares*), 3D maximum tangent modulus using the REBO potential (*red dots*), 3D initial tangent modulus using the REBO potential (*green triangles*), 2D maximum tangent modulus using the Tersoff potential (*blue open squares*), 3D maximum tangent modulus using the Tersoff potential (*cyan stars*), and 3D initial tangent modulus using the Tersoff potential (*pink open circles*). **b** The amount of temperature-dependent elasticity softening: maximum tangent modulus using the REBO potential (*black squares*), initial tangent modulus using the REBO potential (*red dots*), maximum tangent modulus using the Tersoff potential (*green triangles*), and initial tangent modulus using the Tersoff potential (*blue open squares*)

**Fig. 10:**
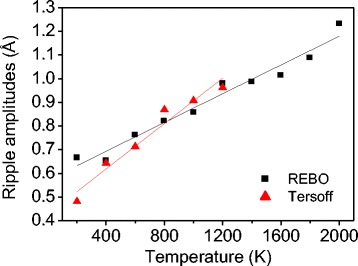
Variation of intrinsic ripple amplitudes of a monolayer graphene as a function of temperature obtained with the REBO potential (*black squares*) and with the Tersoff potential (*red triangles*)

Figure 9a shows the temperature-dependent elasticity calculated from the 2D and 3D MD simulations. For 3D simulations, the initial and maximum values of the tangent modulus are selectively shown. For 2D simulations, the initial and maximum values are the same since elasticity decreases through the entire strain range. For 2D simulations, the elasticity does not change significantly with varying temperature since there is no rippling effect. The slightly decreasing elasticity in 2D is due to the thermal effect on the C–C bond stretching. The decreasing slope is larger for the Tersoff potential (blue open squares) than for the REBO potential (black squares). In 3D simulations, for the REBO and Tersoff potential, both the maximum and initial elasticity decrease almost linearly as the temperature increases. This reduction follows the tendency of increasing out-of-plane displacements shown in Fig. 10, showing a linear increase depending on temperature. The decreasing slope of the Tersoff potential is larger than that of the REBO potential, suggesting that the Tersoff potential is more sensitive to change in temperature. For the Tersoff potential, at 200 K, the maximum and initial elasticity are the same due to the continuous decrease in the tangent modulus as shown in Fig. 8d. These differences between REBO and Tersoff can be explained by change in ripple amplitudes shown in Fig. 10. For the Tersoff potential, the ripple amplitudes increase more sharply than for the REBO potential. In addition, the ripple amplitudes are lower in the low-temperature ranges, resulting in weaker elasticity softening than the REBO potential.

Figure 9b shows the amount of elasticity softening due to rippling, which is calculated by subtracting the 3D values from the 2D results. For the REBO potential, at 300 K, the maximum and initial elasticity are reduced by 7.4 and 9.6 %, respectively. As the temperature increases, the softening increases almost linearly and at 2000 K, elasticity softening reaches 16.5 and 35.3 % for the maximum and initial elasticity, respectively. For the Tersoff potential, the maximum elasticity softening almost agrees with the REBO potential. However, the initial elasticity reduction is smaller and similar to the maximum elasticity softening at low temperature due to the smaller ripple amplitudes. The ripple amplitude at 300 K is reported as 0.6 Å [13], which is between the REBO (0.7 Å) and Tersoff potential (0.5 Å). The optimized Tersoff potential proposed by Lindsay and Broido shows better thermal conductivity since the optimization is focused on the in-plane modes rather than the out-of-plane mode [39]. Good agreement of the phonon dispersion for the in-plane modes may result in reduction of ripple amplitudes, possibly underestimating elasticity softening of graphene. Recently, a new potential has been proposed [41, 42] by Monteverde, Migliorato and Powell, which refers to as MMP potential, and predicted better phonon dispersion of graphene than the Tersoff and REBO potentials [43]. Although the MMP potential may produce more accurate results, it is expected that the trend of the temperature-dependent elasticity softening is similar.

## Conclusions

In this study, the mechanical behavior of the graphene monolayer under a uniaxial tensile test is investigated using MD simulations with a focus on the effect of intrinsic ripples. The simulations reveal that graphene as a 2D material shows nonlinear mechanical behavior even at a small strain, where the response is analogous to entropic elastic behavior such as that of biomaterials [36]. In the graphene monolayer, the entropic contribution is attributed to the intrinsic ripples. Graphene under uniaxial tension undergoes competition of entropic contribution due to the rippling and energetic contribution as a result of the C–C bond stretching. The elastic softening increased almost linearly as temperature increases since ripple amplitudes increase. The study implies that the mechanical properties of graphene such as elasticity can change according to the intrinsic ripple amplitudes, which depend on various environmental conditions such as temperature, van der Waals interactions with a substrate, and the number of graphene layers. The understanding can be useful for strain-based ripple manipulation in graphene engineering. Recently, it has been reported that the controlling bandgap of graphene was possible by the formation of large ripples under compressive strain [43]. Large and regular ripple formation based on compressive strain will be a future work for engineering applications.
